# Emerging role of ETV4 in colorectal cancer: from molecular mechanisms to clinical implications

**DOI:** 10.3389/fonc.2026.1908045

**Published:** 2026-07-15

**Authors:** Yuanbin Liu, Yiyun Wang, Min Huang, Xia Tian, Xiaodong Huang

**Affiliations:** Department of Gastroenterology, Tongren Hospital of Wuhan University (Wuhan Third Hospital), Wuhan, China

**Keywords:** biomarker, colorectal cancer, ETV4, therapeutic target, transcription factor

## Abstract

ETV4 (ETS-transformation-specific variant 4) is a member of the ETS transcription factor family that has been extensively studied for its oncogenic functions in cancers. Here, we summarize the role and mechanisms of ETV4 in cancer biology, with a particular focus on colorectal cancer (CRC), as well as its biomarkers and therapeutic potential. As a transcription factor, ETV4 regulates the expression of target genes by recognizing the GGA(A/T) core conserved sequence. It is frequently overexpressed in pan-cancer, where its overexpression associated with poor prognosis. Upon activation by oncogenic signaling pathways (e.g., MAPK, PI3K/Akt, and WNT), ETV4 transcriptionally regulates downstream genes to promote tumor cell proliferation, invasion, migration, epithelial-mesenchymal transition (EMT), chemoresistance, metabolic reprogramming, and immune evasion. It also form a vicious positive feedback loop that continuously activates oncogenic signaling. In CRC, ETV4 is overexpressed, correlating with advanced disease, lymph node metastasis, and poor prognosis. Upon activation by oncogenic signals, ETV4 directly activate target genes (such as matrix metalloproteinases) to mediate adenoma-to-adenocarcinoma progression, proliferation, EMT, ferroptosis, invasion, metastasis, metabolic reprogramming, and tumor microenvironment remodeling in CRC. ETV4 also forms transcriptional complexes with certain epigenetic factors, such as miRNAs and p300. Moreover, ETV4 is a promising biomarker for CRC diagnosis, prognosis, and adenoma-to-adenocarcinoma progression. Targeting ETV4 or its upstream pathways represents a potential therapeutic strategy for CRC. However, there are still many unresolved issues in current research. For example, the role of ETV4 in immune evasion and tumor microenvironment remodeling remains at the descriptive stage, and the specific mechanisms by which it regulates immune cell recruitment have not yet been elucidated. Future efforts include utilizing multi-omics approaches to elucidate the mechanisms by which ETV4 shapes the CRC microenvironment and exploring the application prospects in tumor immunotherapy.

## 1 Introduction

Colorectal cancer (CRC) is the third most common cancer and the second leading cause of cancer-related deaths worldwide. There are over 1.9 million new cases of CRC annually, with approximately 900,000 deaths, accounting for about 10% of global cancer cases and deaths, respectively (1). Although significant progress has been made in recent years in early screening, surgical treatment, chemoradiotherapy and targeted therapy for CRC, the prognosis for patients with advanced CRC remains poor. Approximately 25% of CRC patients have already developed distant metastases at the time of diagnosis (2), and resistance to standard treatment regimens remain major obstacles in clinical treatment. A thorough understanding of the molecular mechanisms underlying the onset and progression of CRC, as well as the identification of new therapeutic targets and prognostic biomarkers, represents an urgent clinical need and holds significant research interest.

Abnormal activation of transcription factors is one of the key driving events in the onset and progression of CRC. The ETS (E26 transformation-specific) transcription factor family is one of the largest transcription factor families in mammals; to date, nearly 30 members have been identified, and they play an indispensable role in regulating gene expression, determining cell fate, and guiding tissue development (3). Members of this family all contain a highly conserved ETS DNA-binding domain capable of recognizing and binding to the purine-rich DNA sequence 5’-GGA(A/T)-3’, thereby initiating or repressing the transcription of downstream target genes (4). ETS variant transcription factor 4 (ETV4), a member of the ETS family, has emerged as a key oncogenic driver in various types of cancer. As a potent transcription factor, ETV4 regulates multiple tumor characteristics by modulating target genes involved in proliferation, metastasis, metabolic reprogramming, immune evasion, and treatment resistance (5). In recent years, a growing body of research has revealed the abnormal activation of ETV4 in cancer and its role as a tumor-promoting factor, including its oncogenic function in CRC. In CRC, ETV4 is abnormally upregulated and is closely associated with advanced tumor staging, metastasis, poor patient prognosis, as well as with various malignant phenotypes (5, 6). However, there remains a lack of up-to-date systematic summaries and discussions regarding the specific roles of ETV4 in the pathogenesis and progression of CRC. Here, we summarize the multifaceted molecular mechanisms of ETV4 in CRC biology and its specific, context-dependent roles in driving the onset, progression, and treatment resistance of CRC, highlighting its potential value as a relevant biomarker and therapeutic target. We also discussed the multitude of unresolved issues currently facing ETV4 research in CRC and outlined future research directions in this field, with the aim of providing new insights and a theoretical foundation for basic research and clinical translation in CRC.

## 2 Overview and structural characteristics of ETV4

ETV4, also known as PEA3 (polyomavirus enhancer activator 3) or E1AF (E1A enhancer-binding protein), is a key member of the PEA3 subfamily within the typical ETS family transcription factor characterized by a highly conserved ETS DNA-binding domain capable of recognizing the core conserved sequence 5’-GGA(A/T)-3’ in target gene promoters (7, 8). It should be noted that the specificity of ETV4 lies in its preferential recognition of the flanking regions surrounding this core sequence in the target gene promoter, as well as in the specificity of the assembled complex, rather than in the core sequence itself (9). The ETV4 gene is located on human chromosome 17q21 and encodes a transcription factor protein consisting of 484 amino acid residues (6). Its protein structure comprises several functionally distinct domains: the N-terminal transcription activation domain is responsible for recruiting transcription coactivators and the basic transcription machinery; the central ETS DNA-binding domain confers sequence-specific DNA-binding ability; and the C-terminal transcription activation domain (7). While different sources vary in their nomenclature for these domains (e.g., “PEA3-type ETS transcription factor N-terminal domain” versus “N-terminal TAD”), the overall domain architecture is consistent across studies (6). Under normal physiological conditions, ETV4 and other family members precisely regulate key biological processes such as embryonic development, organogenesis, and cell differentiation (6). However, when there are abnormalities in expression levels or transcriptional activity, they often transform from normal developmental regulators into drivers of malignant tumors.

ETV4’s transcriptional activity is regulated by various post-translational modifications (PTMs), including acetylation, SUMOylation, ubiquitination, and phosphorylation (5). For example, phosphorylation mediated by serine/threonine kinases (such as ERK and PKA) significantly enhances ETV4’s transcriptional activity and prolongs its protein half-life; conversely, ubiquitination-mediated degradation catalyzed by E3 ubiquitin ligases (such as COP1) constitutes a negative regulatory mechanism, maintaining a dynamic equilibrium in ETV4 protein levels (10, 11). Mechanistically, ERK directly inhibits the protein-protein interaction between COP1 and ETV4 after phosphorylating ETV4 at Ser73, rather than physically shielding the specific lysine residues targeted by COP1 through steric hindrance (12). Specifically, ETV4 expressions and activity are tightly regulated at multiple levels, including transcriptional induction by several signaling pathways (such as RAS/MAPK, PI3K/AKT, WNT/β-catenin, and hepatocyte growth factor (HGF)/c-MET), PTMs, and non-coding RNA-mediated silencing (7, 13–16). The RAS/MAPK signaling pathway is a major upstream regulator of ETV4, whereas Capicua (CIC), acting as a transcriptional repressor and tumor suppressor downstream MAPK, suppresses ETV4 expression (17, 18). In addition, the FGF signaling pathway also induces ETV4 expression and regulates chromatin status (19, 20). Recent studies have revealed that ETV4 acts as a key transcriptional hub, integrating inputs from multiple oncogenic signaling pathways, including but not limited to WNT/β-catenin, MAPK/MEK/ERK, hypoxia-inducible factor signaling, and p300-dependent epigenetic regulation—to form a complex upstream-downstream regulatory network (14, 21–23). Some upstream transcription factors, such as SOX2 and p63, have also been demonstrated to regulate ETV4 gene expression (24). This multi-level, multidimensional regulatory model enables ETV4 to respond flexibly to different cellular microenvironments and stress conditions, thereby performing diverse biological functions (**Table 1**).

**Table 1:** Major post-translational modifications and upstream regulatory pathways of ETV4.

References	Factor type	Regulatory factor	Transcription activity direction
(5)	PTMs	Acetylation, SUMOylation, ubiquitination, and phosphorylation	Promotion (acetylation/SUMOylation/phosphorylation); Inhibition (ubiquitination)
(7)	Pathways	RAS/MAPK	Promotion
(13)	Pathways	PI3K/AKT	Promotion
(14)	Pathways	WNT/β-catenin	Promotion
(15)	Pathways	HGF/c-MET	Promotion
(15, 19, 20)	Pathways	FGF/FGFR	Promotion
(21)	Pathways	Hypoxia-inducible factor signaling	Promotion
(17, 18)	Transcriptional repressor	Capicua	Inhibition
(24)	Transcription factor	SOX2 and p63	Promotion
(22)	Epigenetic factors	p300	Promotion
(16, 83)	Epigenetic factors	miR-451 and miR-29b	Inhibition
(88)	Epigenetic factors	METTL14	Inhibition

PTMs: post-translational modifications; METTL14: methyltransferase 14.

## 3 Clinical relevance and mechanisms of ETV4 in cancer biology

### 3.1 Pan-cancer expression profile and clinical prognostic value of ETV4

**Figure 1:**
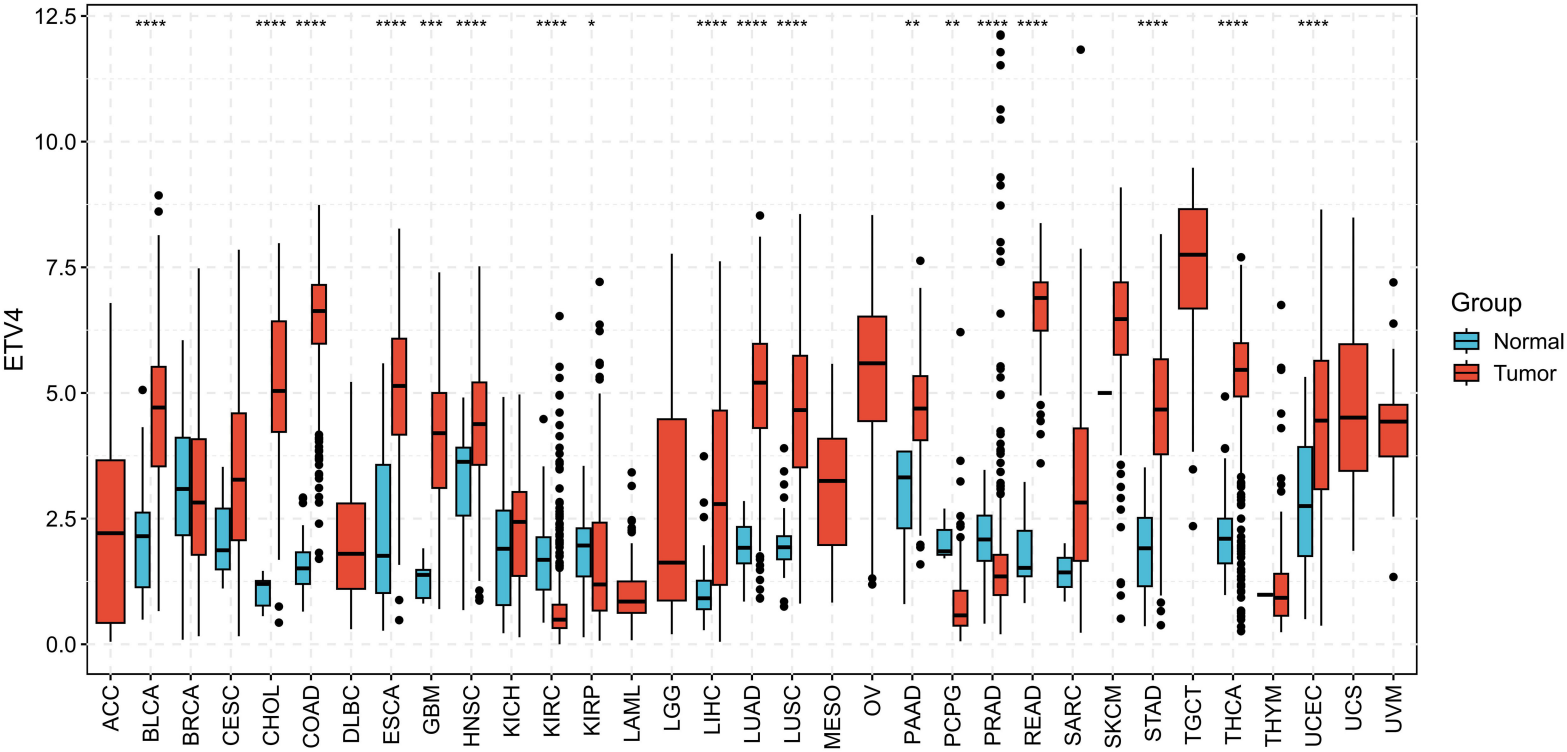
Analysis based on the TCGA data indicates that ETV4 is frequently upregulated in pan-cancer. Asterisks indicate different levels of significance: * P < 0.05, ** P < 0.01, *** P < 0.001, **** P < 0.0001.

ETV4 is abnormally and frequently overexpressed in various types of cancer. Multiple clinical studies and large-scale bioinformatics analyses have shown that ETV4 is significantly associated with overall survival in more than 10 types of cancer, including but not limited to prostate cancer, bladder cancer, gastric cancer, hepatocellular carcinoma (HCC), cholangiocarcinoma, pancreatic cancer, breast cancer, ewing sarcoma, lung adenocarcinoma, and non-small cell lung cancer, and its high expression often portends poorer clinical outcomes (5, 25). For example, gene fusions and overexpression of the ETS family, including ETV4, have been identified in approximately half of Caucasian male prostate cancer patients and TMPRSS2:ETV4 gene fusions define a distinct molecular subtype of prostate cancer (26, 27). ETV4 is significantly upregulated in pancreatic ductal adenocarcinoma tissues and synergistically promote tumor progression with KRAS mutations (28). In breast cancer, ETV4 is particularly highly expressed in the triple-negative subtype and is associated with increased tumor aggressiveness (29). ETV4 is highly expressed in non-small cell lung cancer tissues and is significantly associated with lymph node metastasis and advanced stages (30). However, it is also important to note the heterogeneity in ETV4 expressions in cancers such as prostate and breast cancer; that is, ETV4 expression levels are low in some patients and are not associated with prognosis (5). Pan-cancer analyses reveal that ETV4 participates in the regulation of multiple signaling pathways and is closely associated with tumor molecular subtypes and immune subtypes (25). Analysis based on the TCGA data indicates that ETV4 is frequently upregulated in pan-cancer (**Figure 1**).

### 3.2 ETV4 promotes uncontrolled cancer cell proliferation

ETV4 promotes uncontrolled cancer cell proliferation by directly activating key cell cycle regulatory factors, thereby accelerating the G1/S phase transition. It binds to the promoters of cyclin D1 (CCND1), CCND2, and CCND3 and enhances their transcription (5, 31, 32), which drives cells from the G1 phase into the S phase and further initiates the expression of genes related to DNA replication. In addition, ETV4 can suppress the expression of cell cycle inhibitory factors such as p21 and p27, which inhibit cell cycle progression by blocking CDK activity (33). In multiple malignancies, ETV4 exhibits significant pro-proliferative effects. For example, in prostate cancer, ETV4 overexpression accelerates the transition from the G1 phase to the S phase of the cell cycle by downregulating the expression of the cell cycle inhibitor p21, thereby lifting its inhibition of the CDK2/cyclin E complex and promoting cell proliferation (34). In pancreatic cancer, ETV4 transcriptionally activate the expression of CCND1, promoting the rapid proliferation of pancreatic ductal adenocarcinoma cells (35). Furthermore, in gastrointestinal stromal tumors, ETV4 promotes tumor cell proliferation by inducing CCND1 expression and activating the Wnt/β-catenin signaling pathway (14).

ETV4 also indirectly enhance cell proliferation by inhibiting apoptosis. In glioblastoma, ETV4 suppresses autophagy-dependent apoptosis by transcriptionally activating EMP1 (epithelial membrane protein 1) and maintaining the activity of the PI3K/AKT/mTOR signaling pathway (36). ETV4 directly binds to the EMP1 gene promoter, positively regulating EMP1 expression, an upstream regulator that continuously activates the PI3K/AKT/mTOR pathway—a core survival signaling pathway in cancer cells. ETV4 activates the transcription of long noncoding RNA C2CD4D-AS1, which in turn enhances NEK2 expression by binding a microRNA (miR), miR-3681-3p, thereby promoting the proliferation of lung adenocarcinoma and inhibiting apoptosis (37).

### 3.3 ETV4 mediates epithelial-mesenchymal transition and drives invasion and metastasis

ETV4 downregulates the expression of epithelial markers such as E-cadherin and upregulates the expression of mesenchymal markers such as N-cadherin, vimentin, and Snail (34, 38). Furthermore, ETV4 promotes tumor invasion and metastasis by regulating the expression of matrix metalloproteinases (MMPs), such as MMP1, MMP2, MMP7, MMP9, MMP13, MMP14, and MMP24 (16, 18, 39–42). For example, in esophageal squamous cell carcinoma, the ERK-MAPK signaling pathway-activated ETV4 enhances the expression of MMP2 and MMP9, thereby inhibiting E-cadherin and promoting tumor cell invasion and metastasis (43). In prostate cancer cells, ETV4 overexpression leads to downregulation of E-cadherin and upregulation of N-cadherin, and significantly increases the expression of EMT-specific transcription factors (34). Elevated ETV4 levels in intrahepatic cholangiocarcinoma cells drive EMT by activating the TGF-β1/SMAD signaling pathway (44). *In vitro* experiments indicate that miR-451-targeted activation of ETV4 promotes EMT and tumor progression in non-small cell lung cancer by directly activating MMP13 (16). By targeting MMP13, ETV4 promotes the migration and invasion of breast cancer cells, whereas inhibition of MMP13 disrupts ETV4-induced migration and invasion (40). In addition, angiogenesis is a key feature of tumor metastasis. Recent studies have shown that in HCC, ETV4 upregulate the expression of MMP14, thereby promoting angiogenesis and metastasis (45). The upstream inhibitor of ETV4, CIC, suppresses invasion and metastasis in a lung cancer metastasis model, whereas inactivation of CIC drives ETV4-mediated upregulation of MMP24, which is both necessary and sufficient for metastasis (18).

ETV4 also directly contributes to tumor invasion and metastasis through multiple signaling pathways. The hepatitis B virus X protein upregulates ETV4 by increasing its chromatin accessibility through the enrichment of H3K27ac in the ETV4 gene; upon activation, ETV4 promotes HCC cell migration and invasion by upregulating Dishevelled 2 and activating the Wnt/β-catenin pathway (46). ETV4 transcriptionally activates annexin A2 expression, thereby activating the Wnt/β-catenin signaling pathway and promoting the migration and invasion of HBV-associated HCC (47). In gastrointestinal stromal tumors, ETV4 induces CCND1 expression while simultaneously activating the Wnt/β-catenin pathway, thereby promoting cell invasion (14). Upon activation, the PI3K/AKT pathway activates mTOR, which promotes protein synthesis and provides the energy and material basis for invasion and metastasis. In prostate cancer, overexpression of ETV4 directly activates the PI3K and RAS signaling pathways, which constitute the core regulatory networks governing cell survival and invasion (13). Interestingly, the PI3K/AKT pathway positively regulates ETV4 as an upstream pathway. In clear cell renal cell carcinoma cells, the PI3K-AKT pathway induces ETV4 expression, and ETV4 directly binds to the FOS Like 1 (FOSL1) promoter to increase FOSL1 expression in a PI3K-AKT-dependent manner, leading to metastasis (48). ETV4 upregulates kinesin family protein 2A expression, an activator of the PI3K/AKT signaling pathway, thereby inducing migration and invasion of gastric cancer cells in an AKT signaling pathway-dependent manner (49). TGF-β signaling is another important pathway involved in tumor metastasis. Studies have shown that ETV4 binds to the B3GNT3 promoter and activates its transcription, thereby activating the TGF-β signaling pathway and promoting HCC cell migration, invasion, and EMT (50). Earlier evidence suggests that the HGF/c-MET signaling pathway, as an upstream pathway of ETV4, is also involved in cancer cell invasion, possibly through the Rho/ROCK pathway and by activating MMP genes (51, 52).

### 3.4 ETV4 regulates tumor metabolic reprogramming to induce stemness and malignancy phenotypes

ETV4 participates in the regulation of tumor metabolic reprogramming, primarily by modulating related pathways and the expression of metabolism-related genes. In cholangiocarcinoma, ETV4 promotes glycolysis by regulating the TGF-β signaling pathway, thereby increasing glucose uptake, lactate production, and extracellular acidification rates, which provide energy for tumor growth (53). ETV4 is a key transcription factor regulating the Warburg effect. By directly transcriptionally activating the expression of key glycolytic enzymes such as hexokinase 2 and lactate dehydrogenase A, ETV4 promotes glucose uptake and lactate production (54). In breast cancer, ETV4 knockdown significantly downregulates the expression of multiple glycolysis-related genes, including solute carrier family 12 member 1 (SLC2A1), ALDOA, GAPDH, PGK1, ENO1, and PDK1, and inhibits extracellular acidification rates and lactate production; mechanistic studies indicate that ETV4 induces stemness by promoting glycolysis and transcriptionally enhancing CXCR4 (C-X-C Motif Chemokine Receptor 4) expression to activate the sonic-Hedgehog signaling pathway (54). Clinical sample analysis shows that ETV4 expression levels are positively correlated with glycolytic signaling in breast cancer and hepatocellular carcinoma tissues (54). In addition, ETV4 also transcriptionally activates SLC12A5, a protein that indirectly enhances the glycolytic capacity and resistance to ferroptosis of breast cancer cells by regulating ion homeostasis, thereby promoting breast cancer cell proliferation, migration, and invasion (55). ETV4 directly binds to the NSUN2 promoter to activate its transcription; NSUN2 subsequently regulates the expression of pyruvate kinase M2, thereby promoting aerobic glycolysis and hypopharyngeal squamous cell carcinoma cell proliferation and invasion, while inhibiting apoptosis (56). Recent studies have shown that M2 macrophage exosomes highly express ETV4 and are internalized by HCC cells. ETV4 in exosomes promotes HCC cell proliferation, glycolysis, and maintenance of stem-like properties by directly binding to and transcriptionally activating SULT2B1 (57). In summary, ETV4 reshapes the tumor metabolic landscape, thereby mediating and amplifying oncogenic signals.

### 3.5 ETV4 reshapes the tumor microenvironment to promote immune evasion and metastasis

ETV4 reshapes the TME by regulating the expression of cytokines, chemokines, and immune checkpoint molecules, thereby promoting tumor immune evasion and progression. In HCC, ETV4 serves as one of the core transcription factors involved in a malignant transformation axis characterized by the secretion of secreted phosphoprotein 1 (SPP1) to recruit and polarize SPP1⁺ macrophages, thereby creating an M2-like TME with angiogenic and immunosuppressive properties (58). Another study found FGF19/FGFR4- and HGF/c-MET-activated ETV4 upregulates the expression of programmed death-ligand 1 (PD-L1) and CCL2, promotes the infiltration of tumor-associated macrophages (TAMs) and myeloid-derived suppressor cells, and creates an immunosuppressive microenvironment, thereby facilitating tumor metastasis (15). In lung cancer, oncogenic KRAS mutations activate the MEK-ERK signaling pathway, thereby upregulating ETV4 expression; ETV4 directly binds to the PD-L1 promoter, thereby transcriptionally activating PD-L1 and promoting immune evasion (59). ETV4 also activates Aurora kinase A, which further regulates the JAK2/STAT3 signaling pathway to indirectly promote PD-L1 expression, thereby suppressing CD8+ T cell activity and mediating immune evasion in lung cancer (60). In melanoma, ETV4 directly mediates tumor immune evasion by transcriptionally activating PD-L1. High ETV4 expression is negatively correlated with immune infiltration, thereby attenuating T-cell-mediated tumor killing and predicting a poor response to PD-1 inhibitor therapy (61). Another recent study found that ETV4 directly upregulates CD47 and activates the macrophage immune checkpoint axis, the CD47-SIRPα (signal regulatory protein α) signaling, thereby inhibiting macrophage phagocytosis and promoting immune suppression in melanoma (62). In addition to PD-L1, a signaling pathway originating in the extracellular matrix activates the PD-L1/PD-L2 super-enhancer via the integrin/BRAF/TAK1/ERK/ETV4 axis, driving the constitutive expression of PD-L1 and PD-L2, thereby mediating tumor immune evasion (63). Furthermore, in bladder cancer, ETV4 also mediates the infiltration of tumor-associated neutrophils (TANs), promotes lymphangiogenesis, and facilitates lymphatic metastasis, suggesting that ETV4 plays a universal role in the remodeling of the TME (64).

**Figure 2:**
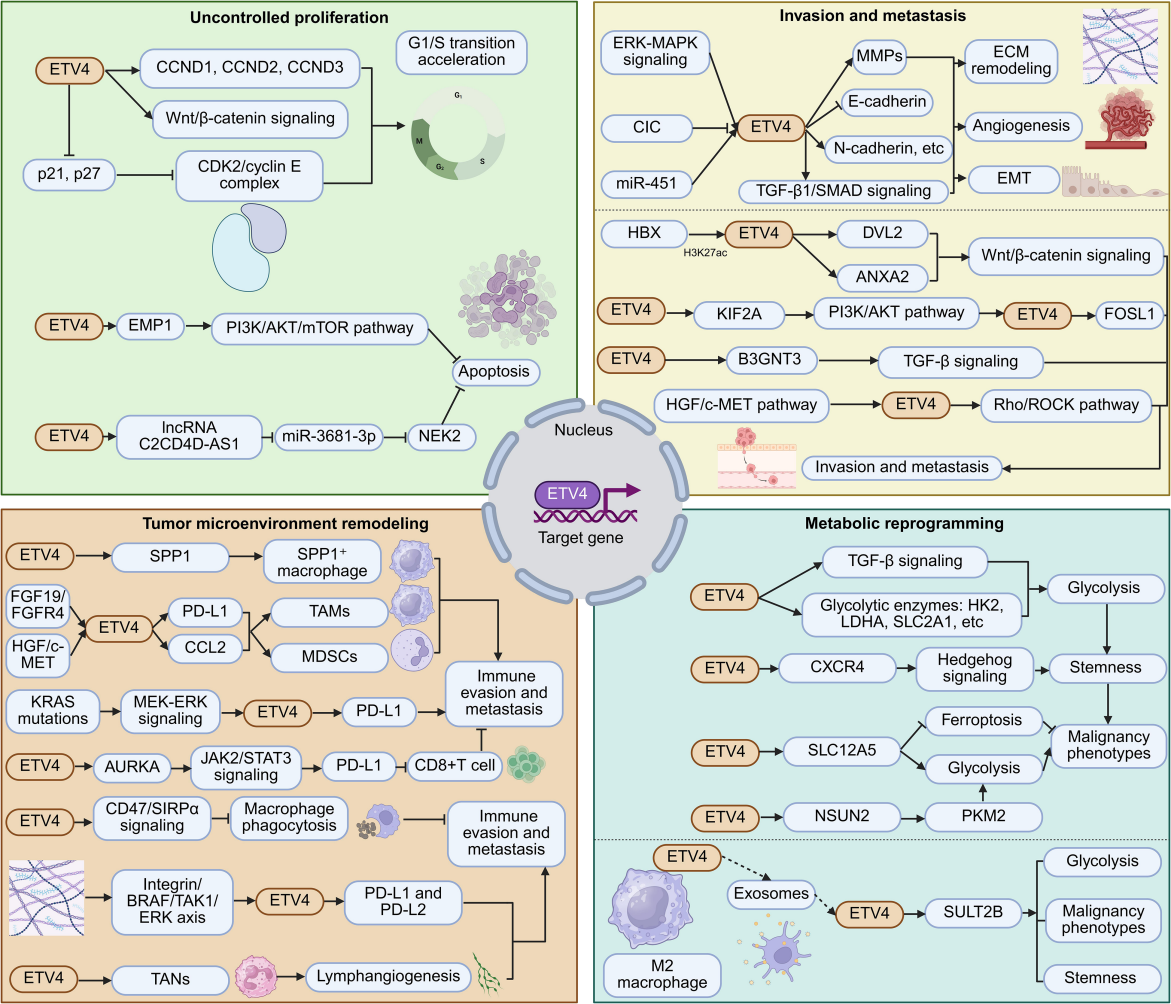
The role of ETV4 in cancer biology. Its upstream and downstream signaling pathways, target genes, mechanisms, and regulation of malignant phenotypes are summarized in the respective quadrants.

Overall, ETV4 plays a significant role in cancer biology by regulating various signaling pathways and target genes (**Figure 2**).

## 4 Roles and mechanisms of ETV4 in CRC

### 4.1 ETV4 is overexpressed in CRC and predicts poor outcomes

Numerous studies have demonstrated that ETV4 is significantly overexpressed in CRC tissues (25, 65–70). Immunohistochemical analysis revealed that 62 of 100 CRC tissue samples were ETV4-positive, whereas normal colorectal tissue showed no or only weak staining (65). RT-PCR analysis revealed that ETV4 mRNA expression was detected in all 10 cases of liver metastases from CRC (65). High expressions of ETV4 are closely associated with lymph node metastasis, depth of tumor invasion, recurrence, and clinical staging in CRC (65, 68, 70). More importantly, ETV4-high CRC patients have significantly lower overall survival and disease-free survival rates (65, 67, 71). Bioinformatics analysis suggests that ETV4 is associated with stemness score and immune subtypes of CRC (25, 69). Analysis of the TCGA-COAD co-expression network revealed that ETV4 was positively correlated with RNF43, CDK18, E2F1, and SLC35C2, suggesting that ETV4 may act in concert with these genes to promote CRC cell proliferation and activation of the WNT/β-catenin pathway. Conversely, ETV4 was negatively correlated with CARD16, FAS, and MED11, suggesting that high ETV4 expression may promote tumor progression by inhibiting apoptosis and immune responses.

The CMS classification divides CRC into four subtypes: CMS1 (MSI-immune), CMS2 (classic), CMS3 (metabolic), and CMS4 (mesenchymal). To date, there remains a lack of research linking ETV4 to CMS. A spatial transcriptomics analysis identified a potential regulatory role of DCN in the transcriptional activity of ETV4 at the tumor-stroma interface in CMS2 tumors and confirmed that this regulation persists in liver metastases (72); this is currently the only study that directly links ETV4 to CMS classification. Here we propose a speculative, yet-to-be-validated hypothesis that high ETV4 expression correlates closely with the mesenchymal phenotype of CMS4. This subtype is characterized by EMT, stromal infiltration, TGF-β activation, and immunosuppression—features that align with the functions regulated by ETV4. In addition, CMS4-type CRC is often associated with KRAS/BRAF mutations, leading to sustained activation of the MAPK pathway. ETV4 may be a potential transcriptional hub linking MAPK signaling abnormalities to the CMS4 mesenchymal phenotype, but a direct association requires further experimental validation.

### 4.2 ETV4 promotes the initiation of CRC and adenoma-to-adenocarcinoma progression

Research has shown that ETV4 plays a significant role in the adenoma-to-adenocarcinoma transition in CRC. Gene chip and TCGA database expression analyses of paired adenoma and adenocarcinoma samples revealed that ETV4 is one of the most highly expressed genes in adenocarcinoma samples, suggesting that ETV4 is involved in early events of CRC and promote adenoma-to-adenocarcinoma transition (73). Another cDNA microarray study consistently demonstrated that ETV4 is overexpressed in colorectal adenomas and early-stage invasive cancers compared to normal tissue, suggesting that ETV4 plays a significant role in the early development of CRC (74). MMPs play a key role in the onset and progression of CRC. ETV4 expression is significantly elevated in adenoma and early-stage CRC tissues and is positively correlated with the expression of MMP-1, MMP-7, COX-2 (cyclooxygenase-2), and iNOS (inducible nitric oxide synthase), suggesting that ETV4 drive the early stages of CRC by synergistically upregulating these invasion- and inflammation-related genes (75, 76).

### 4.3 ETV4 promotes CRC cell proliferation, migration, and invasion

ETV4 knockdown reduced CRC cell proliferation and invasion (77); whereas c-kit/MEK/ERK pathway-activated ETV4 promoted the invasion of colorectal mucinous adenocarcinoma (78). Deletion of the transcription repressor CIC enhances cell invasion, migration, and proliferation by derepressing ETV4 (17). The canonical WNT/β-catenin pathway plays a critical role in the proliferation, migration, and invasion of CRC cells. Bioinformatics analysis and *in vitro* experiments have confirmed that ETV4 is a downstream target gene of the WNT/β-catenin signaling pathway, and inhibition of this pathway downregulates ETV4 levels (71). ETV4 were positively correlated with the levels of nuclear/total β-catenin, c-Myc, and CCND1 (71). Interestingly, R-spondin 2 inhibits the downstream PKC/ERK signaling cascade in a non-canonical WNT signaling pathway-dependent manner by inducing Fzd7 degradation, leading to the clearance of ETV4 and reduced expression of MMP7 and CD44, thereby suppressing CRC metastasis (79). MMPs critically participate in the migration and invasion of CRC cells. ETV4 and MMP-1 expressions are significantly correlated, and ETV4 knockdown in CRC cells reduces MMP-1 levels and invasion (65). Another study similarly demonstrated that ETV4 knockdown downregulates a range of MMPs and inhibits invasion and metastasis both *in vitro* and *in vivo (*80). MMPs are involved in regulating CRC cell proliferation. MMP1 derived from TAMs activates ETV4 via the PAR1/ERK1/2 pathway, which in turn induces a series of cell cycle protein complexes, including CCND1, significantly promoting the proliferation of CRC cells by accelerating the G1/S transition. Furthermore, ETV4 directly binds to the MMP1 promoter and activate MMP1 transcription, confirming the positive feedback loop of MMP1/ETV4/MMP1 (81).

Furthermore, ETV4 regulate EMT and angiogenesis to promote metastasis. In CRC cells, ETV4 overexpression downregulates the epithelial marker E-cadherin while upregulating the mesenchymal markers N-cadherin, vimentin, and Twist1. It should be noted that ETV4 primarily regulates and promotes EMT through indirect signaling pathways, rather than acting directly as an upstream activator of classical EMT transcription factors. ETV4 directly binds to and activates LOXL2 expression, forming a protein complex that mediates the demethylation of the NID1 promoter, thereby upregulating NID1 expression and activating the downstream ERK signaling pathway, which in turn drives EMT and metastasis (82). In CRC, ETV4 is positively correlated with CCND1 expression, while miR-29b is negatively correlated with ETV4 (70). ETV4 upregulates the expression of EMT- and angiogenesis-related factors such as MMP-2, MMP-9, vimentin, and vascular endothelial growth factor, and downregulate E-cadherin and thrombospondin-1 via the miR-29b/ETV4/ERK/EGFR axis, thereby promoting the migration, invasion, EMT, and angiogenesis of CRC cells (83). In addition, ETV4 transcriptionally activates HES1 to promote Stat3 phosphorylation, leading to EMT, proliferation, migration, and invasion (84).

**Figure 3:**
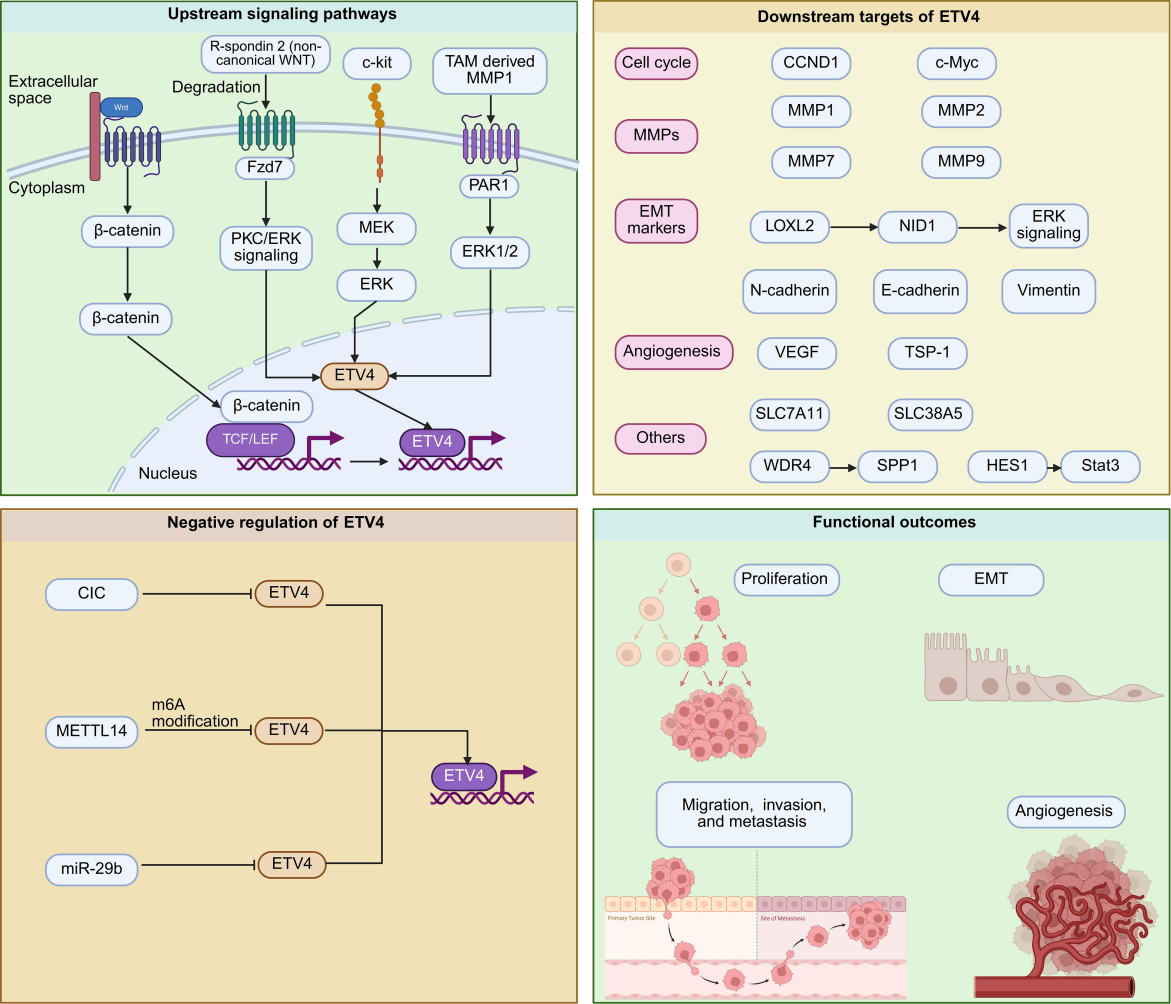
ETV4 promotes CRC cell proliferation, migration, and invasion. The four quadrants summarize the upstream signaling pathways, negative regulation, downstream targets, and functional outcomes of ETV4 in regulating these processes.

ETV4 regulate proliferation and migration through other mechanisms. Integrated single-cell transcriptomic and epigenetic analyses revealed that ETV4 is significantly upregulated in CRC as a ferroptosis regulator (indicating that ETV4 may act as an oncogene to promote CRC progression by inhibiting ferroptosis), and its H3K27ac signal is significantly higher than that in normal colonic tissue (85). ETV4 forms a transcriptional complex with histone acetyltransferase p300, which enhances WDR4 expression through H3K27ac-dependent chromatin remodeling, thereby stabilizing SPP1 mRNA, inhibiting autophagy, and promoting tumor proliferation and metastasis (22). ETV4 inhibits ferroptosis by activating SLC7A11, whereas silencing ETV4 inhibits CRC cell proliferation, colony formation, and migration (86). Another study found that ETV4 acts as a transcription factor by directly binding on the SLC38A5 promoter to activate its expression, thereby promoting the proliferation, migration, and invasion of CRC cells (87). METTL14 (methyltransferase 14) mediates the degradation of ETV4 mRNA by reducing its stability through m6A modification, thereby inducing apoptosis and ferroptosis in CRC cells and inhibiting proliferation (88) (**Figure 3**).

### 4.4 ETV4 promotes metabolic reprogramming and remodels TME

**Figure 4:**
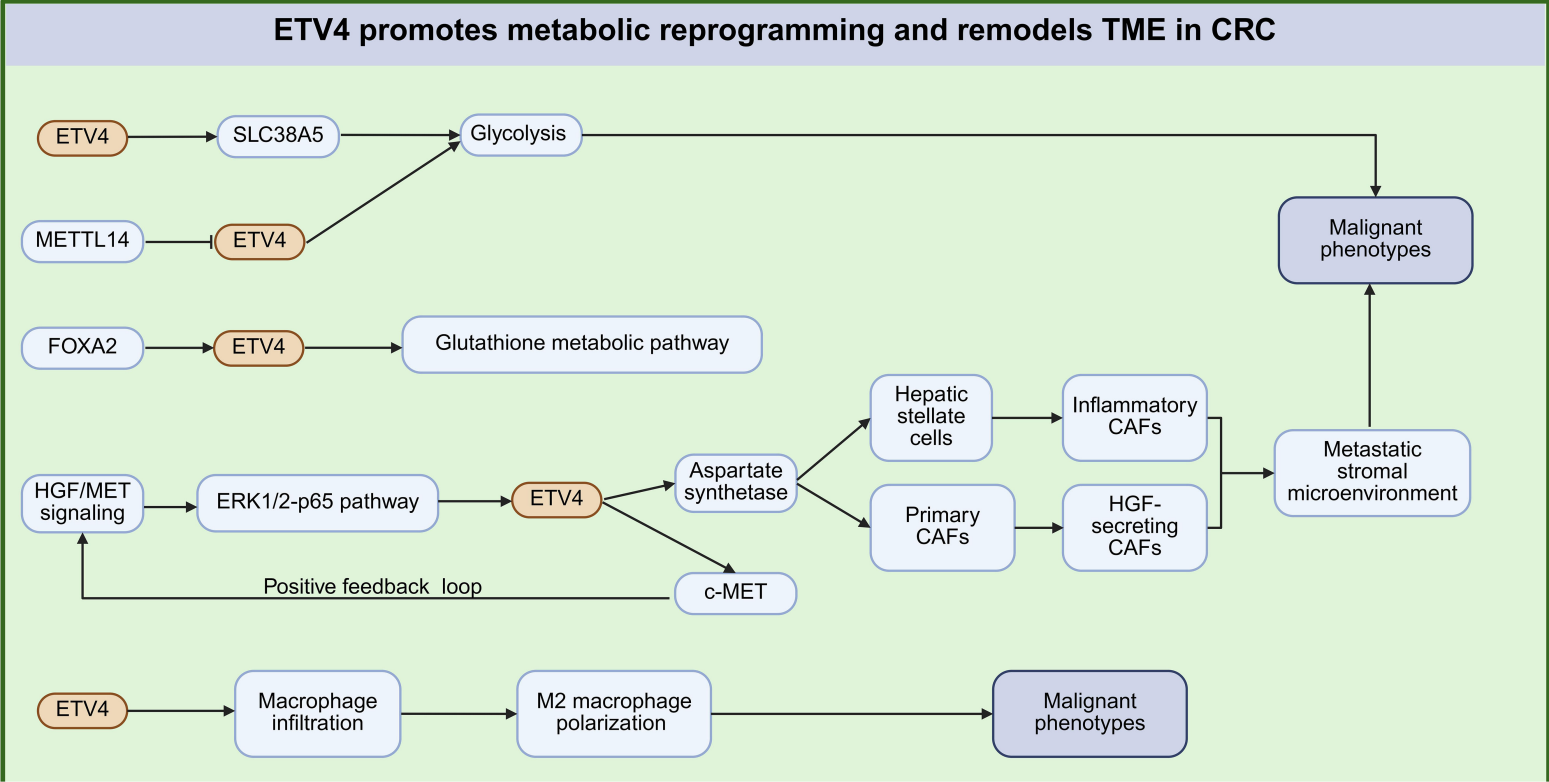
ETV4 promotes metabolic reprogramming and remodels TME in CRC.

ETV4 transcriptionally activates the amino acid transporter SLC38A5, and SLC38A5 deficiency inhibits glycolytic activity in CRC cells, while also suppressing cell proliferation, migration, and oxaliplatin resistance (87). METTL14-mediated downregulation of ETV4 protein expression via m6A modification inhibits glycolysis in CRC cells (88). A recent study found that FOXA2 transcriptionally activated-ETV4 promotes CRC progression by activating the glutathione metabolic pathway, forming a FOXA2/ETV4/glutathione metabolic regulatory axis (89). Another study found that HGF/MET signaling induces ETV4 expression via the ERK1/2-p65 pathway, and ETV4 directly transcriptionally activates MET and aspartate synthetase, forming a positive feedback amplification loop and promoting the malignant phenotype of CRC cells. Importantly, elevated intracellular asparagine acts as a paracrine signal that induces hepatic stellate cells to become activated in a manner resembling inflammatory cancer-associated fibroblasts (CAFs) and promotes the polarization of primary CAFs into inflammatory, HGF-secreting CAFs, thereby reprogramming the metastatic stromal microenvironment of CRC (90). Bioinformatics analysis has identified ETV4 as one of the genes associated with EMT in CRC and has shown enrichment in fatty acid metabolism and AMPK pathway (91); however, studies directly examine the role of ETV4 in regulating fatty acid metabolism in CRC remain limited. Integrated transcriptomic and single-cell analyses revealed that ETV4 is significantly associated with the pattern of macrophage infiltration in CRC. Flow cytometry and immunohistochemistry confirmed that ETV4 is directly involved in the polarization of immunosuppressive M2 macrophages in the TME (71) (**Figure 4**).

### 4.5 Biomarkers and therapeutic potential of ETV4 in CRC

A microarray study of paired adenoma-adenocarcinoma samples showed that ETV4 was among the genes most significantly upregulated in the adenocarcinoma samples. Therefore, ETV4 shows great potential as a biomarker for adenoma-adenocarcinoma progression (73). Furthermore, given that ETV4 is also one of the genes showing the most significant upregulation in the early stages of CRC, it also serve as a potential biomarker (74, 76). A bioinformatics analysis identified ETV4 as one of the hub genes distinguishing normal tissue from CRC (both early-stage and advanced-stage) through weighted correlation network analysis, and it demonstrated strong predictive performance (92). Recent evidence directly indicates that the combined prognostic power of ETV4, LOXL2, and NID1 is superior to that of any single biomarker, suggesting that ETV4 holds promise as a member of a combined signature gene predictive biomarker panel (82). Another study identified ETV4, CLDN1, and CA2 from a series of EMT biomarkers as the most promising genetic surrogates, demonstrating the highest predictive accuracy and discriminatory power (93). Therefore, ETV4 represents a diagnostic biomarker for CRC as one of the core hub genes in the gene network (70, 94). ETV4 expression was further increased in lymph node-positive cases, suggesting that it is a potential marker of CRC aggressiveness and metastasis (68). ETV4 is located downstream of the K-ras signaling pathway, suggesting that it serve as a marker of activated K-ras pathways and a biomarker for predicting response to anti-EGFR therapy (66). ETV4 also functions as a prognostic biomarker for CRC, promoting disease progression by reshaping the immunosuppressive microenvironment (71).

Given the critical role of ETV4 in CRC progression, targeting ETV4 has emerged as a potential new strategy for CRC treatment. Currently, research on ETV4-targeted therapies for cancer remains in the preclinical stage. Gene therapy is a key strategy for suppressing ETV4 expression. RNA interference technologies, such as small interfering RNA (siRNA) and short hairpin RNA (shRNA), specifically silence ETV4 expression, thereby inhibiting tumor cell proliferation, migration, and invasion. *In vitro* and *in vivo* studies have shown that siRNA/shRNA-mediated ETV4 silencing significantly inhibits the growth and metastasis of CRC cells and sensitizes cells to chemotherapies (82, 84, 86, 87). Furthermore, CRISPR/Cas9 gene editing technology can be used to knock out the ETV4 gene, permanently suppressing ETV4 expression and providing a new avenue for ETV4-targeted therapy.

Upstream interventions targeting ETV4 also offer new avenues for research; for example, METTL14, CIC, and miR-29b have been shown in experimental models to suppress ETV4 expression (17, 83, 88). Therefore, targeting these upstream molecules, at least in part, halts the progression of CRC by inhibiting ETV4. Phosphorylation of ETV4 at Ser73 by ERK kinase blocks the ubiquitination and degradation of ETV4 in CRC, leading to its overexpression (12). Since ERK activation stabilizes the ETV4 protein, ERK inhibitors (U0126) inhibit EMT and angiogenesis in CRC by reducing ETV4 levels (83). The FAK inhibitor (PF573228) or ERK inhibitor (U0126) reduces p-ERK1/2 levels, which inhibit ETV4 expression and, consequently, suppress EMT and metastasis in CRC (82). Another ERK1/2 inhibitor (ASTX029) suppressed HGF-induced ETV4 expression, and the combination of an inhibitor targeting the upstream HGF/MET pathway and an asparaginase targeting the downstream pathway of ETV4 demonstrated significantly superior tumor suppression effects in CRC compared to monotherapy (90).

Since ETV4 primarily exerts its effects by binding to the promoter regions of target genes, small-molecule inhibitors can be designed to block ETV4’s DNA-binding activity, thereby inhibiting the transcriptional activation of downstream target genes. Currently, several small-molecule compounds capable of inhibiting ETS transcription factor activity have been identified in other cancers (95); however, no specific small-molecule inhibitors targeting ETV4 have yet been developed. Further research is needed to screen for and develop highly effective, low-toxicity, specific ETV4 inhibitors. Furthermore, in non-small cell lung cancer cells, ETV4 knockdown enhanced aumolertinib-induced apoptosis and G2/M arrest and synergistically inhibited tumor growth *in vivo*, suggesting that ETV4 intervention serve as a combination therapy strategy to overcome acquired chemotherapy resistance (96).

Overall, ETV4 harbors significant potential as a biomarker (**Table 2**) and therapeutic agent (**Table 3**) in CRC.

**Table 2:** Biomarkers potential of ETV4 in CRC.

References	Types of biomarkers	Description
(73)	Adenoma-adenocarcinoma progression	One of the most expressed in adenocarcinoma
(74)	Early colorectal tumors	Over five times higher than in normal tissue
(76)	Early colorectal tumors	Overexpressed and related to overexpression of COX-2 and MMP-7
(92)	CRC	One of the core genes identified by weighted correlation network analysis
(82)	CRC prognosis	Combined ETV4, LOXL2, and NID1 marker panels are more reliable than using any single marker alone
(93)	CRC	Top-ranked by data-driven reference
(70)	CRC	Overexpressed and positively correlated with CCND1
(94)	CRC	High diagnostic value
(68)	CRC invasiveness and metastasis	Overexpressed and further increased in lymphoid node involvement
(66)	Response to anti-EGFR therapy	Downstream of the K-ras signaling
(71)	CRC prognosis	reshapes TME

CRC, colorectal cancer; COX-2, cyclooxygenase-2; MMP7, matrix metalloproteinase-7; ETV4, ETS-transformation-specific variant 4; LOXL2, lysyl oxidase-like 2; CCND1, cyclin D1; TME, tumor microenvironment.

**Table 3:** Therapeutic potential of ETV4 in CRC.

Major references	Treatments	Mechanisms
(82, 84, 86, 87)	siRNA/shRNA	Specifically silence ETV4 expression
(88)	METTL14	m6A modification
(17)	Capicua	Transcription repression
(83)	miR-29b	Directly bind to and degrade ETV4 mRNA
(83)	ERK inhibitors (U0126)	Inhibiting ERK-mediated phosphorylation of ETV4
(82)	FAK inhibitor (PF573228) or ERK inhibitor (U0126)	Inhibition of FAK or ERK1/2 phosphorylation
(90)	ERK1/2 inhibitor (ASTX029)	Inhibition of p65 phosphorylation

CRC, colorectal cancer; ETV4, ETS-transformation-specific variant 4; METTL14, methyltransferase 14.

## 5 Research gaps and future directions for ETV4 in CRC

There remain many unresolved issues regarding the study of ETV4 in CRC. For example, the signaling networks in which ETV4 is involved are highly complex, but there is a lack of comprehensive mechanistic studies on its specific role in the cross-regulation of multiple signaling pathways. Research on the role of ETV4 in immune evasion and TME remodeling remains at a descriptive stage, with insufficient understanding of the underlying mechanisms. Existing studies have found that high expression of ETV4 is significantly associated with the polarization of immunosuppressive M2 macrophages; however, the specific mechanisms by which it regulates the recruitment and functional transformation of immune cells have not yet been fully elucidated. Research on ETV4-mediated metabolic reprogramming and its relationship to treatment resistance has so far been limited to a few metabolic pathways. The clinical value of ETV4 as a diagnostic and prognostic marker requires further validation; and more effective targeted therapeutic strategies for ETV4 still need to be developed.

In the future, efforts should focus on fully leveraging single-cell RNA sequencing and spatial transcriptomics to map the expression patterns of ETV4 across different cell types and microenvironmental compartments, thereby elucidating its spatiotemporal dynamics within the heterogeneous landscape of CRC. *In vivo* immune models can be utilized to investigate the molecular mechanisms by which ETV4 regulates the immune microenvironment, and metabolomics can be employed to systematically identify the metabolic pathways regulated by ETV4. From a translational perspective, preclinical models can be used to explore the tumor-suppressive effects of combining ETV4 inhibition with immunotherapy or metabolic therapy on CRC tumor growth. Currently, targeted therapies for ETV4 still face challenges. As a transcription factor, ETV4 lacks a typical enzyme-binding pocket, making the development of traditional small-molecule inhibitors difficult. Future research directions include developing inhibitors that target ETV4 protein-protein interactions, exploring targeting strategies for upstream regulators of ETV4, and personalized immunotherapy regimens based on ETV4 expression levels.

## 6 Conclusions

ETV4 plays a multidimensional and multilevel regulatory role in cancer biology, particularly in the progression of CRC. ETV4 activates downstream target genes through direct transcriptional regulation and forms regulatory networks with various pathways. ETV4 plays a crucial role in cancer biology by regulating tumor cell proliferation, EMT, metastasis, metabolic reprogramming, and TME remodeling. In CRC, ETV4 is abnormally overexpressed, and its expression levels are closely associated with staging, metastasis, and prognosis. ETV4 promotes the adenoma-to-adenocarcinoma transition, progression, metabolic reprogramming, and TME remodeling of CRC through various molecular mechanisms. Studies have demonstrated the potential of ETV4 as a diagnostic and prognostic biomarker for CRC. Approaches targeting ETV4 expressions, such as RNA interference and upstream pathway inhibitors, have shown tumor-suppressive effects in experimental models. Further research is needed to investigate the molecular mechanisms and biomarkers and therapeutic potential of ETV4 in CRC.
